# Early post-marketing safety profile of resmetirom in FAERS: an exploratory assessment of statin co-reporting

**DOI:** 10.3389/fphar.2026.1895524

**Published:** 2026-08-20

**Authors:** Yongjian Liu, Jiaru Chen, Hao Zhong, Yige Yu, Shuzhen Liu, Yingyu Yu

**Affiliations:** 1 Department of Pharmacy, Huizhou Central People’s Hospital, Huizhou, China; 2 Department of Pharmacy, Affiliated Hospital of Guangdong Medical University, Zhanjiang, China; 3 Department of Pharmacy, The Third Affiliated Hospital of Sun Yat-sen University, Guangzhou, China; 4 Department of Pharmacy, The First Affiliated Hospital of Wenzhou Medical University, Wenzhou, China; 5 Department of Pharmacy, Shantou Central Hospital, Shantou, China

**Keywords:** disproportionality analysis, drug–drug interaction, FDA adverse event reporting system, metabolic dysfunction-associated steatohepatitis, pharmacovigilance, resmetirom, statins

## Abstract

**Background:**

Resmetirom is the first thyroid hormone receptor-beta agonist approved in the United States for adults with non-cirrhotic metabolic dysfunction-associated steatohepatitis (MASH) and moderate-to-advanced fibrosis. Dyslipidemia and cardiovascular risk are common in MASH, making statin co-use clinically relevant.

**Aim:**

This study aims to characterize early post-marketing adverse event reporting for resmetirom in the FDA Adverse Event Reporting System (FAERS) and explore resmetirom–statin co-reporting.

**Methods:**

Cleaned and deduplicated FAERS reports from 2024 Q1 to 2026 Q1 were analyzed. Positive preferred-term (PT) signals required at least three reports and concordant positivity for the reporting odds ratio, proportional reporting ratio, and Bayesian information component. Reporter-type sensitivity analysis was restricted to healthcare professional (HCP) reports. Exploratory resmetirom–statin analyses used a suspect-anchored definition and prespecified hepatic, gallbladder-related, and muscle-related outcomes.

**Results:**

Among 3,155,054 unique FAERS cases, 1,407 were included in the resmetirom single-drug analysis and 267 met the primary resmetirom–statin co-reporting definition. Reports were concentrated in gastrointestinal, hepatobiliary, investigation, and skin-related system organ classes. The leading positive PTs were diarrhea, nausea, pruritus, alanine aminotransferase increased, and aspartate aminotransferase increased. The findings were compatible with the FDA label and remained similar in the reporter-type sensitivity analysis. Unlabeled terms among the top 20 signals included weight decreased and abdominal discomfort, which require cautious interpretation. Exploratory DDI analysis did not identify a consistent interaction-specific signal for the prespecified hepatic, gallbladder-related, or muscle-related outcomes. Rhabdomyolysis/myopathy was based on five co-reports; the odds ratio was elevated, whereas Omega025 was negative.

**Conclusion:**

Early FAERS data indicate a resmetirom reporting spectrum mainly concordant with the FDA label. Unlabeled non-specific PTs, including weight decreased and abdominal discomfort, require cautious interpretation. Resmetirom-statin co-reporting did not show a stable interaction signal.

## 1 Introduction

Metabolic dysfunction-associated steatohepatitis (MASH), previously referred to as non-alcoholic steatohepatitis, is a liver manifestation of cardiometabolic disease (Younossi et al., 2016; Rinella et al., 2023b; Rinella et al., 2023a). Patients with MASH frequently experience obesity, dyslipidemia, type 2 diabetes, and cardiovascular risk factors, and lipid-lowering drugs are often used in the same clinical setting in which resmetirom is now prescribed (Targher et al., 2016; Rinella et al., 2023b; U.S. Food and Drug Administration, 2024). Resmetirom, a selective thyroid hormone receptor-beta agonist, is the first drug approved in the United States for adults with non-cirrhotic MASH with moderate-to-advanced liver fibrosis (Harrison et al., 2019; Harrison et al., 2023; Harrison et al., 2024). Its clinical introduction raises two post-marketing questions: whether early real-world reporting follows the prescribing information and whether statin co-reporting identifies additional safety concerns.

The resmetirom label describes gastrointestinal adverse reactions, pruritus, dizziness, hepatic laboratory abnormalities, hepatotoxicity, and gallbladder-related events. It also provides statin dose recommendations because resmetirom can increase exposure to several statins (U.S. Food and Drug Administration, 2024). These statements are clinically important but are derived mainly from pre-approval trials and pharmacokinetic evidence. Spontaneous reporting systems cannot establish incidence or causality, but they can identify disproportionate adverse event reporting soon after market entry, especially for newly approved medicines with limited long-term real-world data (Bate and Evans, 2009; Harpaz et al., 2012; Sakaeda et al., 2013; U.S. Food and Drug Administration, 2026).

Drug–drug interaction (DDI) analyses in spontaneous reporting databases are sensitive to design choices (Norén et al., 2008; Jiao et al., 2024). Concomitant drugs may indicate comorbidity rather than causality, and confounding by indication is substantial in MASH because statin use is itself a marker of cardiometabolic risk (Newman et al., 2019; Chalasani et al., 2021; Pastori et al., 2022; Rinella et al., 2023b). We, therefore, characterized resmetirom adverse event reporting in a single-drug analysis and then examined resmetirom–statin co-reporting as an exploratory signal-generating analysis, not as causal interaction testing.

Using FAERS reports from 2024 Q1 to 2026 Q1, we evaluated the resmetirom single-drug adverse event spectrum, annotated whether prominent PTs were described in the FDA label, assessed reporter-type sensitivity, and summarized prespecified hepatic, gallbladder-related, and muscle-related outcomes in resmetirom–statin co-reports.

## 2 Methods

### 2.1 Data source and study period

We conducted a retrospective pharmacovigilance study using FAERS (Sakaeda et al., 2013; U.S. Food and Drug Administration, 2026). Cleaned and deduplicated FAERS quarterly extract files available through 2026 Q1 were analyzed. This time window was selected because resmetirom received US approval in March 2024; therefore, reports before 2024 Q1 were excluded (Harrison et al., 2024; U.S. Food and Drug Administration, 2024). Case-level data were linked across the DEMO, DRUG, REAC, and OUTC tables by case identifier. Duplicate DEMO records were removed using the FDA-recommended approach. “PRIMARYID,” “CASEID,” and “FDA_DT” were extracted; records were sorted by “CASEID,” “FDA_DT,” and “PRIMARYID”; for repeated “CASEID” values, the record with the latest “FDA_DT” was retained; when both “CASEID” and “FDA_DT” were identical, the record with the largest “PRIMARYID” was retained. The retained case identifiers were then used to keep linked DRUG, REAC, and OUTC records (U.S. Food and Drug Administration, 2026). Because FAERS contains publicly available de-identified reports, ethics approval and informed consent were not required.

### 2.2 Drug identification and analytic groups

Resmetirom reports were identified by searching both the “drugname” and “prod_ai” fields using the drug names “resmetirom,” “Rezdiffra,” “MGL-3196,”and “MGL3196” (Harrison et al., 2019; U.S. Food and Drug Administration, 2024). Statins were identified from the same two DRUG-table fields using generic names and major brand or combination-brand names (Supplementary Table S1). In FAERS, drug role codes include primary suspect (PS), secondary suspect (SS), concomitant (C), and interacting (I) roles. For the resmetirom single-drug analysis, resmetirom was restricted to PS reports. For the exploratory co-reporting analysis, resmetirom was defined as drug A and statins as drug B. The primary co-reporting definition was suspect-anchored: A + B cases required resmetirom and a statin in the same case, with at least one of the two drugs coded as PS or SS; A-only cases required resmetirom without a statin, with resmetirom coded as PS or SS; and B-only cases required a statin without resmetirom, with the statin coded as PS or SS. This definition was chosen as a compromise between crude co-reporting and a more restrictive dual-suspect definition. Crude co-reporting may include incidental background medications, whereas requiring both drugs to be coded as suspect drugs may exclude plausible interaction reports in which one drug was recorded as concomitant. The suspect-anchored definition therefore prioritized interpretability while retaining potential co-reporting situations in which one of the two drugs was not coded as PS or SS (Norén et al., 2008; Fusaroli et al., 2024; Jiao et al., 2024). These strata were designed for spontaneous-report co-reporting surveillance and were not intended to represent clinically comparable treatment cohorts.

### 2.3 Case characteristics and serious outcomes

Report characteristics were summarized for the resmetirom single-drug analysis set and for the A + B, A-only, and B-only co-reporting groups. Variables included age availability, age, sex availability, sex, weight availability, weight, number of co-reported drugs, reporter country, reporter type, report year, and serious outcomes. Age was derived from AGE and AGE_COD, converted to years, and retained when between 0 and 120 years. Healthcare professional (HCP) reporters were defined as physicians (MD), pharmacists (PH), or other health professionals (HP); non-healthcare professional reporters were defined as consumers (CN) or lawyers (LW). Serious outcomes were derived from OUTC codes and included death (DE), hospitalization (HO), life-threatening event (LT), disability (DS), congenital anomaly (CA), required intervention (RI), and other serious outcome (OT). OUTC categories were not mutually exclusive.

### 2.4 Single-drug disproportionality analysis

PTs from the REAC table were used as adverse-event terms and coded according to MedDRA version 28.1. For each PT, a two-by-two table compared reports in the resmetirom single-drug analysis with all other FAERS reports in the same study period (Supplementary Table S2). Disproportionality was assessed using the reporting odds ratio (ROR), proportional reporting ratio (PRR), and information component (IC) (Bate et al., 1998; Evans et al., 2001; Van Puijenbroek et al., 2002; Harpaz et al., 2012; Fusaroli et al., 2026). Formulas and positivity thresholds are shown in Supplementary Table S3. Positive PT signals required at least three reports and simultaneous positivity for ROR (lower 95% CI > 1), PRR (PRR ≥2 and chi-square ≥4), and BCPNN/IC (IC_025_ > 0). Prominent PTs were annotated as label-consistent or not explicitly mentioned according to the FDA prescribing information (U.S. Food and Drug Administration, 2024). PTs were mapped to their primary MedDRA system organ class (SOC) using MedDRA version 28.1 primary SOC information (Brown et al., 1999). As a supplementary macroscopic analysis, ROR, PRR, and IC were also calculated at the primary SOC level using the same case-level background and positivity thresholds as the PT-level analysis (Supplementary Table S4) (Brown et al., 1999).

Following recent causal-inference guidance for disproportionality analysis, these metrics were interpreted as measures of drug-event co-reporting rather than causal effect estimates (Fusaroli et al., 2026). Recent causal inference frameworks utilizing directed acyclic graphs emphasize that disproportionality can diverge from causation because of confounding, collider structures, measurement error, and reporting bias and that causal assumptions should be explicit when restrictions are designed or signals are interpreted (Fusaroli et al., 2026). We used PS restriction and reporter-type sensitivity analysis as bias-assessment tools, not as procedures that remove residual confounding.

### 2.5 Reporter-type sensitivity analysis

Reporter-type sensitivity analysis was restricted to reports submitted by healthcare professionals. Both the resmetirom single-drug numerator and the FAERS background denominator were restricted to HCP reports, preserving the same disproportionality framework. The HCP-restricted signal spectrum was compared with the all-reporter spectrum for the leading PTs to assess whether the main resmetirom signals were driven by non-professional reports.

### 2.6 Time-to-onset analysis

Time to onset (TTO) was calculated as the interval between the earliest recorded resmetirom start date and the event onset date. Partial dates were handled by fixed midpoint imputation: six-digit year–month entries were assigned to the 15th day of the month, and four-digit year-only entries were assigned to 1 July. Intervals from 0 to 3,650 days were considered valid. Because TTO availability was limited and date resolution varied across reports, TTO was summarized only as a descriptive supplementary analysis and was not used for causal, subgroup, or interaction inference (Van Holle et al., 2012; Sauzet et al., 2013).

### 2.7 Exploratory resmetirom–statin co-reporting analysis

Prespecified DDI outcome groups were focused hepatic injury, severe hepatic disorders, gallstone-related disorders, and rhabdomyolysis/myopathy (Supplementary Table S5). Focused hepatic injury was a custom cluster derived from severe hepatic injury and hepatic cholestasis/jaundice PTs. Severe hepatic disorders were identified using narrow terms from the Drug-related hepatic disorders—severe events only SMQ. Gallstone-related disorders were identified using the Gallstone-related disorders SMQ. Rhabdomyolysis/myopathy was identified using narrow terms from the Rhabdomyolysis/myopathy SMQ (Brown et al., 1999; MedDRA Maintenance And Support Services Organization, 2025).

Outcome selection was based on the FDA label, known statin safety concerns, mechanistic plausibility, and MedDRA/SMQ groupings. Hepatic injury outcomes were included because the prescribing information describes hepatotoxicity and hepatic laboratory abnormalities. The focused hepatic injury cluster was selected as the primary hepatic outcome to capture clinically meaningful liver injury while reducing contamination from broad MASH backgrounds or chronic liver disease terms. The severe hepatic disorders SMQ was retained as a standardized supportive hepatic outcome. Gallstone-related disorders were included because gallbladder-related adverse reactions are described in the prescribing information and can be operationalized using a specific MedDRA SMQ. Rhabdomyolysis/myopathy was included because the label provides statin dose recommendations during co-administration, reflecting increased exposure to several statins; muscle toxicity is a clinically relevant statin exposure-related safety concern.

For each outcome, event counts were summarized in the A + B, A-only, B-only, and neither groups, and odds ratios were used to compare A + B with A-only reports. Interaction-oriented measures included omega shrinkage statistics and additive interaction indices (Norén et al., 2008; Jiao et al., 2024). Omega is a shrinkage observed-to-expected statistic for drug–drug–event co-reporting under an additive expectation; Omega025 is its lower 95% credibility bound. Omega025 values greater than zero were interpreted as evidence supporting excess co-reporting for the drug–drug–event combination. Omega025 values below zero indicated no stable evidence of excess co-reporting, not a protective effect. Formulas for the DDI metrics are shown in Supplementary Table S3. All DDI metrics were interpreted as unadjusted co-reporting measures rather than adjusted estimates of pharmacological interaction. Sparse results were interpreted cautiously, particularly when event counts were small or metrics were discordant.

### 2.8 Statistical analysis

Analyses were performed in R version 4.5.3 (R Core Team, 2026) using data.table version 1.18.2.1 (Barrett et al., 2026), ggplot2 version 4.0.2 (Wickham, 2016), openxlsx version 4.2.8.1 (Schauberger and Walker, 2025), officer version 0.7.5 (Gohel et al., 2026), and flextable version 0.9.11 (Gohel and Skintzos, 2026). Comparisons of TTO availability across report year, report quarter, reporter type, and serious outcome status were performed using Pearson’s chi-square test when all expected cell counts were at least 5. Fisher’s exact test was used for sparse 2 × 2 tables, whereas a chi-square test with 20,000 Monte Carlo replicates was used for larger sparse tables. Continuous variables were summarized as medians with interquartile ranges and categorical variables as counts and percentages. Disproportionality analyses used case-level deduplicated reports. Zero cells in two-by-two tables were handled with a continuity correction. All analyses were descriptive and signal-generating.

## 3 Results

### 3.1 Study population and analysis sets

**FIGURE 1:**
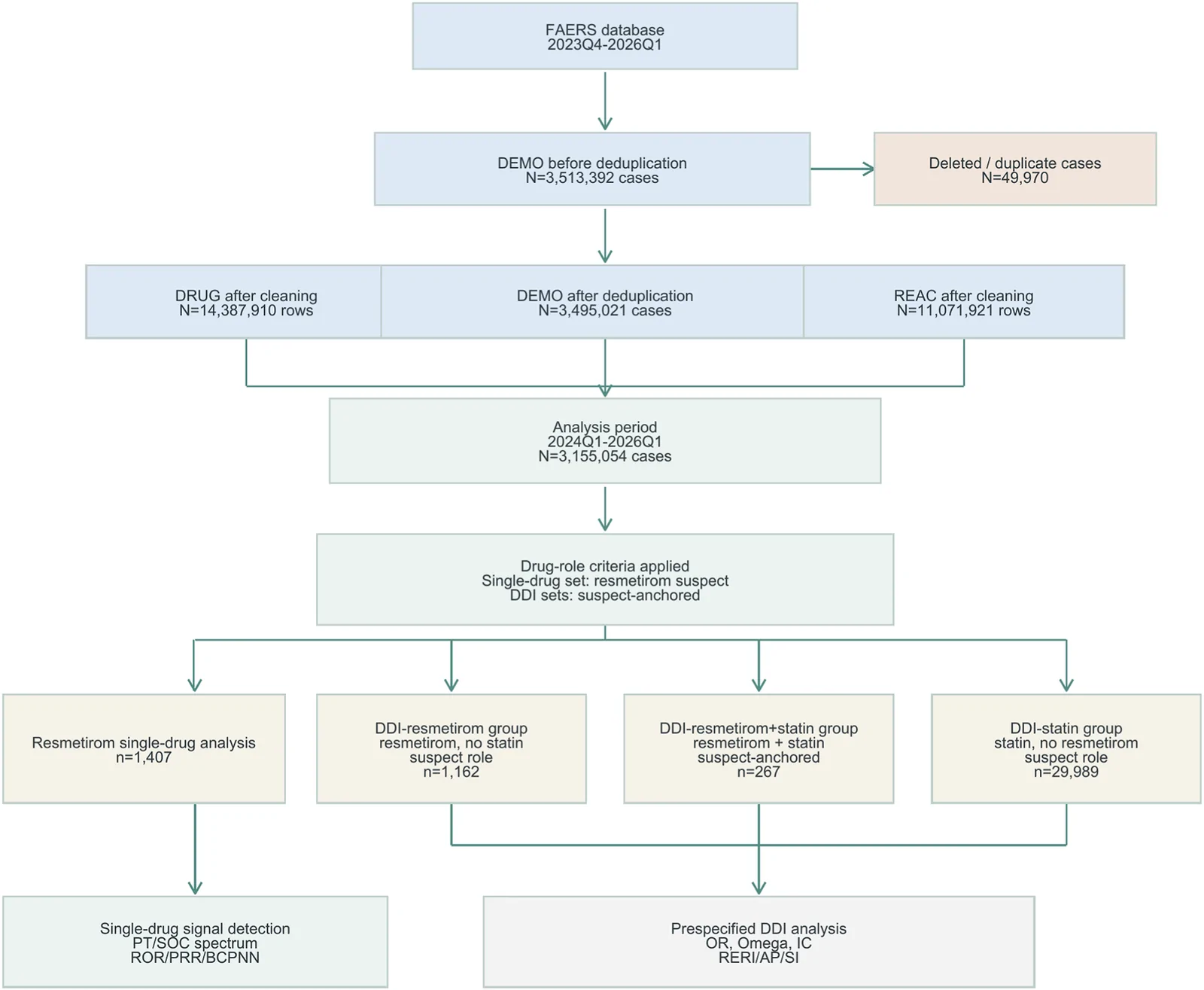
FAERS cleaning, exposure-group definition, and analysis workflow. The flowchart shows FAERS cleaning, case deduplication, analysis-period restriction, and construction of the resmetirom single-drug analysis set and the three DDI groups. Suspect role refers to primary or secondary suspect coding as specified in the Methods. DDI, drug–drug interaction.

After FAERS cleaning and deduplication, the 2024 Q1 to 2026 Q1 analysis window contained 3,155,054 unique case reports. Resmetirom was reported in 1,519 cases with any drug role, including 1,407 reports used for the resmetirom single-drug analysis and 1,429 reports in which resmetirom was coded as PS or SS. Statins were reported in 159,844 cases with any role, including 18,532 PS reports and 29,998 PS/SS reports. Overall, 286 cases co-reported resmetirom and a statin, and 267 met the prespecified primary co-reporting definition in which either resmetirom or a statin was coded as PS or SS (Figure 1). Given the recent U.S. approval of resmetirom in March 2024, the 1,407 single-drug reports and 267 resmetirom–statin co-reports represent an early but analyzable post-marketing reporting volume.

The resmetirom single-drug analysis set included 1,407 cases. Age was available for 148 cases (10.5%), with a median age of 64.0 years (interquartile range, 54.0–70.0). All cases in the resmetirom single-drug analysis were reported from the United States. Serious outcomes were recorded in 419 cases (29.8%), including death in 65 cases (4.6%) and hospitalization in 87 cases (6.2%). In the primary co-reporting analysis, the DDI-resmetirom + statin group contained 267 cases, the DDI-resmetirom group 1,162 cases and the DDI-statin group 29,989 cases. Any serious outcome was reported in 118 (44.2%), 308 (26.5%), and 27,337 (91.2%) cases in these three groups, respectively. The co-medication burden also differed markedly across groups. The median number of co-reported drugs was 1.0 in the resmetirom single-drug analysis and 10.0 in the DDI-resmetirom + statin group, indicating greater case complexity in co-reports (Table 1). The high proportion of serious outcomes in the DDI-statin group indicates a different reporting structure and should be considered when interpreting co-reporting comparisons (Table 1).

**TABLE 1:** Characteristics of the four analysis groups.

Characteristic	Resmetirom	DDI-resmetirom	DDI-resmetirom + statin	DDI-statin
Cases, n	1,407	1,162	267	29,989
Age				
Age available	148 (10.5%)	120 (10.3%)	43 (16.1%)	22,355 (74.5%)
Age, years, median [IQR]	64.0 [54.0, 70.0]	61.0 [52.0, 68.2]	68.0 [58.5, 72.0]	67.0 [59.0, 76.0]
Sex				
Female	32 (2.3%)	38 (3.3%)	11 (4.1%)	13,139 (43.8%)
Male	25 (1.8%)	21 (1.8%)	6 (2.2%)	12,378 (41.3%)
Unknown sex	1,350 (95.9%)	1,103 (94.9%)	250 (93.6%)	4,472 (14.9%)
Weight				
Weight available	51 (3.6%)	37 (3.2%)	23 (8.6%)	10,706 (35.7%)
Weight, kg, median [IQR]	90.7 [78.5, 107.5]	89.5 [79.4, 107.1]	84.2 [77.5, 100.1]	75.0 [63.5, 88.0]
Co-reported drugs, median [IQR]	1.0 [1.0, 6.0]	1.0 [1.0, 2.0]	10.0 [6.0, 15.0]	5.0 [2.0, 10.0]
Report country				
United States	1,407 (100.0%)	1,162 (100.0%)	267 (100.0%)	5,476 (18.3%)
United Kingdom	—	—	—	6,313 (21.1%)
Canada	—	—	—	4,384 (14.6%)
Reporter type				
Top reporter type	CN, 553 (39.3%)	CN, 455 (39.2%)	CN, 103 (38.6%)	MD, 10,834 (36.1%)
Healthcare professional reporter	703 (50.0%)	580 (49.9%)	134 (50.2%)	20,525 (68.4%)
Non-healthcare professional reporter	553 (39.3%)	455 (39.2%)	103 (38.6%)	6,599 (22.0%)
Reporter type missing/other	151 (10.7%)	127 (10.9%)	30 (11.2%)	2,865 (9.6%)
Report year				
2024	250 (17.8%)	203 (17.5%)	49 (18.4%)	12,651 (42.2%)
2025	846 (60.1%)	703 (60.5%)	157 (58.8%)	13,678 (45.6%)
2026	311 (22.1%)	256 (22.0%)	61 (22.8%)	3,660 (12.2%)
Serious outcome				
Non-serious outcome	988 (70.2%)	854 (73.5%)	149 (55.8%)	2,652 (8.8%)
Any serious outcome	419 (29.8%)	308 (26.5%)	118 (44.2%)	27,337 (91.2%)
Death	65 (4.6%)	39 (3.4%)	26 (9.7%)	1,835 (6.1%)
Hospitalization	87 (6.2%)	58 (5.0%)	30 (11.2%)	10,401 (34.7%)
Life-threatening	8 (0.6%)	4 (0.3%)	4 (1.5%)	2,369 (7.9%)
Disability	7 (0.5%)	4 (0.3%)	3 (1.1%)	2,178 (7.3%)
Required intervention	4 (0.3%)	2 (0.2%)	2 (0.7%)	104 (0.3%)
Other serious	388 (27.6%)	286 (24.6%)	109 (40.8%)	21,787 (72.6%)

Values are n (%) unless otherwise indicated. A + B denotes reports co-reporting resmetirom and a statin, with at least one of the two drugs recorded as primary suspect (PS) or secondary suspect (SS). A only and B only denote the DDI-resmetirom and DDI-statin comparator groups, respectively. Reporter type follows FAERS, occupation codes; CN, consumer; MD, physician; PH, pharmacist; HP, other health professional; LW, lawyer. Serious outcomes were derived from OUTC, records; OUTC categories are not mutually exclusive.

### 3.2 SOC-level distribution of resmetirom reports

**FIGURE 2:**
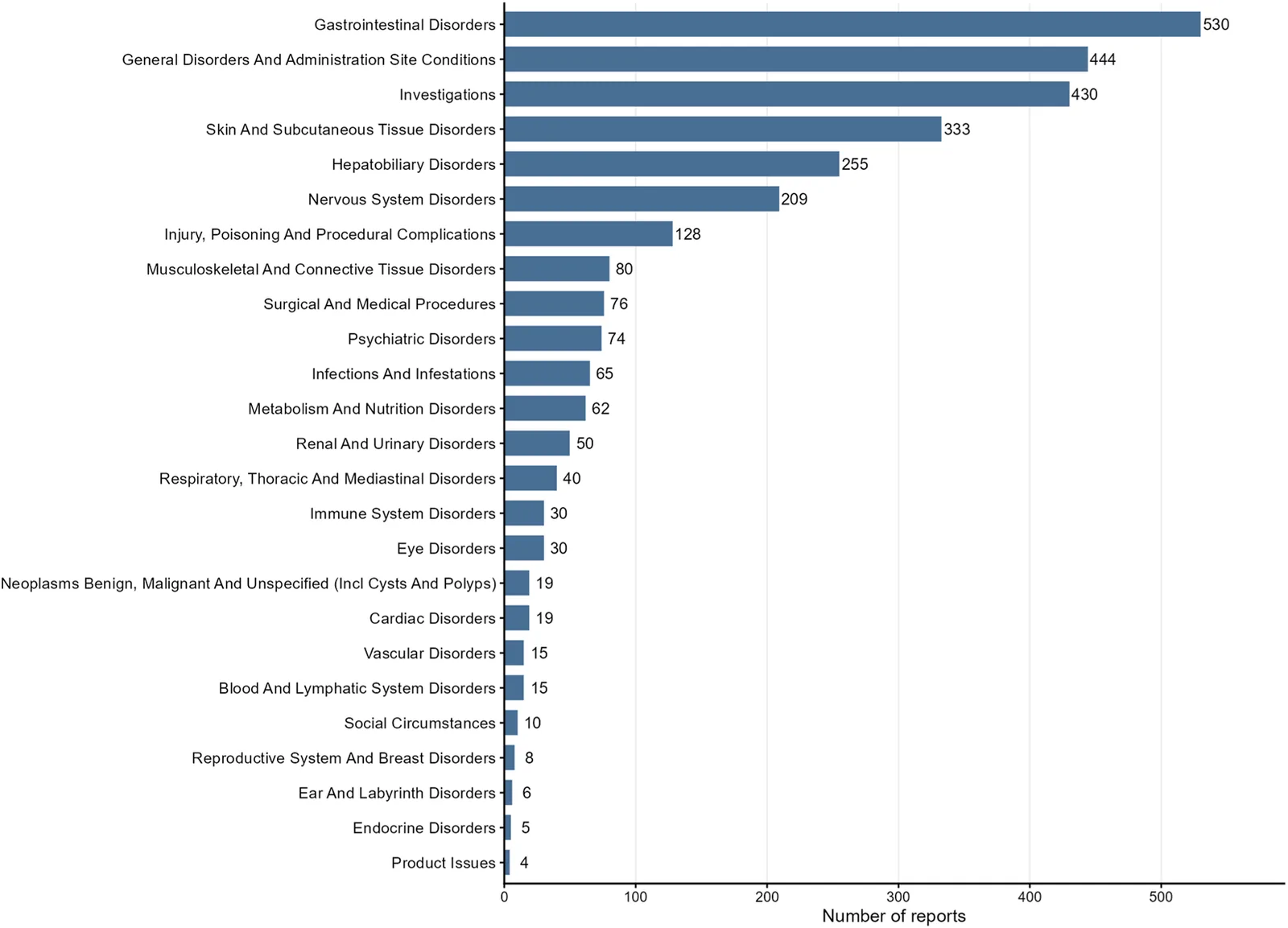
SOC-level report counts in the resmetirom single-drug analysis. Horizontal bars show the number of reports assigned to each primary MedDRA system organ class in the resmetirom single-drug analysis. SOC terms were mapped using MedDRA 28.1 primary SOC information.

At the SOC level, reports in the resmetirom single-drug analysis were concentrated in gastrointestinal disorders, general disorders and administration site conditions, investigations, skin and subcutaneous tissue disorders, and hepatobiliary disorders (Figure 2). Gastrointestinal disorders accounted for 530 reports, followed by general disorders and administration site conditions (444 reports), investigations (430 reports), skin and subcutaneous tissue disorders (333 reports), and hepatobiliary disorders (255 reports). The reported events clustered predominantly within gastrointestinal symptoms, laboratory abnormalities, hepatobiliary manifestations, and pruritus/skin-related events. In the supplementary SOC-level disproportionality analysis, 4 of 28 primary SOCs met all three positivity criteria: Gastrointestinal Disorders, Investigations, Skin And Subcutaneous Tissue Disorders, and Hepatobiliary Disorders (Supplementary Table S4). No other primary SOC met any of the three positivity criteria.

### 3.3 Positive PT signal spectrum of resmetirom

**FIGURE 3:**
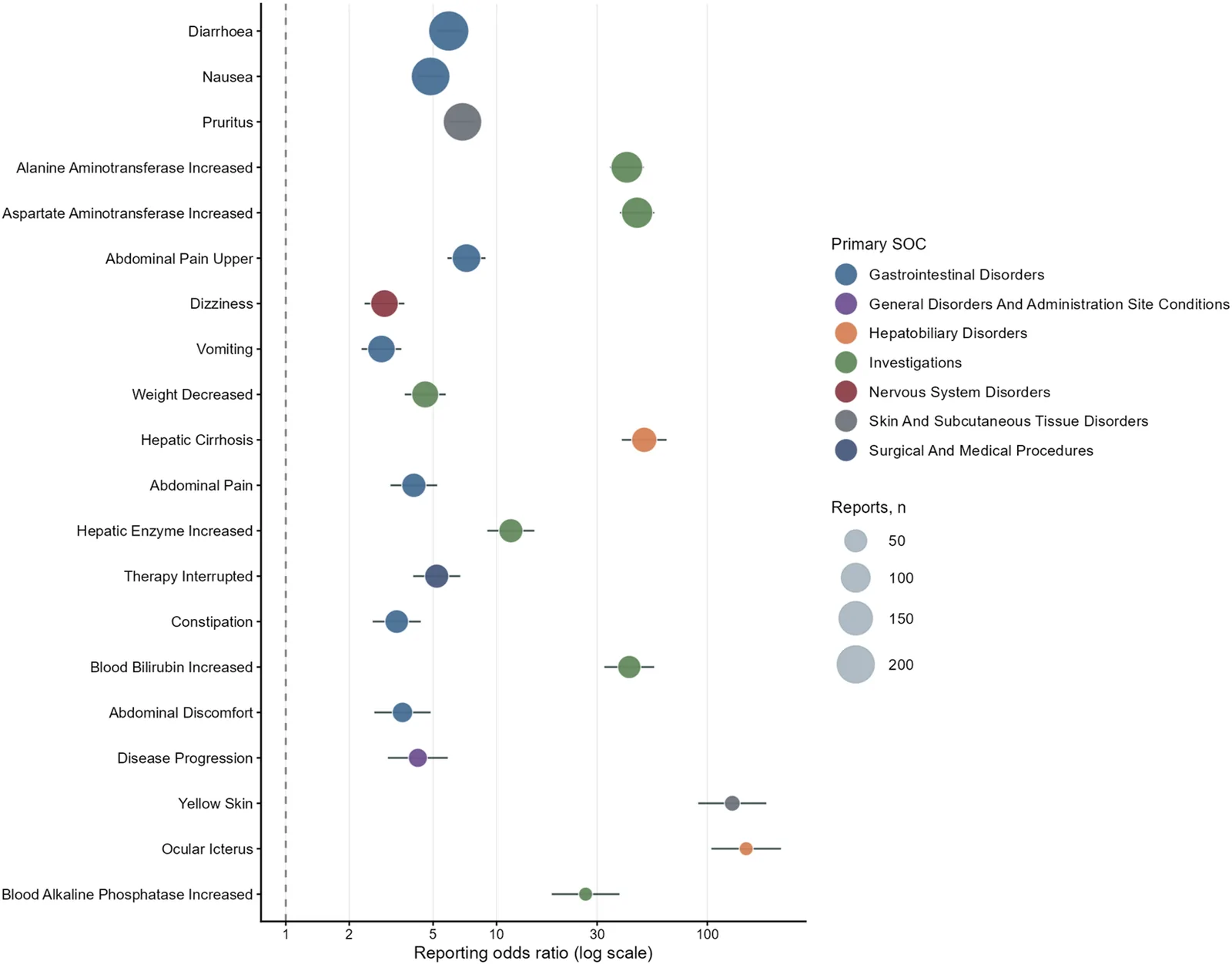
Top 20 positive PT signals in the resmetirom single-drug analysis. The bubble forest plot shows the top 20 positive preferred terms in the resmetirom single-drug analysis. Points indicate RORs, horizontal lines indicate 95% confidence intervals, and bubble size indicates the number of reports. Positive PTs required at least three reports and simultaneous positivity for ROR (lower 95% CI > 1), PRR (PRR ≥2 and chi-square ≥4), and BCPNN/IC (IC_025_ > 0).

Using the prespecified three-algorithm positivity definition, positive PT signals in the resmetirom single-drug analysis comprised mainly gastrointestinal, pruritus-related, and hepatobiliary/laboratory terms (Figure 3; Table 2). The most frequently reported positive PTs were diarrhea (243 cases; ROR, 5.93; 95% CI, 5.17–6.81), nausea (218 cases; ROR, 4.86; 95% CI, 4.21–5.62), pruritus (216 cases; ROR, 6.89; 95% CI, 5.96–7.97), alanine aminotransferase increased (127 cases; ROR, 41.52; 95% CI, 34.55–49.90), and aspartate aminotransferase increased (122 cases; ROR, 46.44; 95% CI, 38.51–56.01). Other label-consistent signals included abdominal pain upper, vomiting, constipation, blood bilirubin increased, jaundice, blood alkaline phosphatase increased, hepatic pain, and liver function test increased (U.S. Food and Drug Administration, 2024).

**TABLE 2:** Top 20 positive PT signals in the resmetirom single-drug analysis. Positive PTs were defined as preferred terms with at least three reports and simultaneous positivity for ROR, PRR, and BCPNN/IC criteria.

PT	SOC	Reports, n	ROR (95% CI)	PRR	IC_025_
Diarrhea	Gastrointestinal disorders	243	5.93 (5.17–6.81)	5.08	2.15
Nausea	Gastrointestinal disorders	218	4.86 (4.21–5.62)	4.27	1.89
Pruritus	Skin and subcutaneous tissue disorders	216	6.89 (5.96–7.97)	5.99	2.37
Alanine aminotransferase increased	Investigations	127	41.52 (34.55–49.90)	37.86	4.78
Aspartate aminotransferase increased	Investigations	122	46.44 (38.51–56.01)	42.50	4.91
Abdominal pain upper	Gastrointestinal disorders	96	7.20 (5.85–8.86)	6.78	2.43
Dizziness	Nervous system disorders	87	2.94 (2.37–3.65)	2.82	1.18
Vomiting	Gastrointestinal disorders	87	2.84 (2.29–3.53)	2.73	1.13
Weight decreased*	Investigations	82	4.59 (3.67–5.74)	4.38	1.79
Hepatic cirrhosis*	Hepatobiliary disorders	69	50.19 (39.31–64.09)	47.78	4.80
Abdominal pain	Gastrointestinal disorders	62	4.05 (3.14–5.23)	3.92	1.58
Hepatic enzyme increased	Investigations	61	11.69 (9.04–15.12)	11.23	3.01
Therapy interrupted*	Surgical and Medical procedures	61	5.20 (4.02–6.72)	5.02	1.92
Constipation	Gastrointestinal disorders	58	3.36 (2.58–4.37)	3.26	1.31
Blood bilirubin increased	Investigations	55	42.64 (32.48–55.96)	41.01	4.51
Abdominal discomfort*	Gastrointestinal disorders	42	3.58 (2.63–4.87)	3.50	1.33
Disease progression*	General disorders and administration site conditions	37	4.23 (3.05–5.87)	4.15	1.53
Yellow skin	Skin and subcutaneous tissue disorders	30	131.35 (90.57–190.49)	128.57	4.84
Ocular Icterus	Hepatobiliary disorders	29	152.90 (104.60–223.50)	149.77	4.86
Blood alkaline phosphatase increased	Investigations	29	26.46 (18.28–38.31)	25.94	3.65

Positive PTs were defined as preferred terms with at least three reports and simultaneous positivity for ROR, PRR, and BCPNN/IC criteria. An asterisk (*) indicates PTs not explicitly mentioned as adverse reactions in the FDA label.

Several high-ranking PTs were not explicitly mentioned as adverse reactions in the FDA label. Among the top 20 positive signals, these included weight decreased, hepatic cirrhosis, therapy interrupted, abdominal discomfort, and disease progression (Table 2). In the complete positive PT table, additional unlabeled terms included unevaluable event, metabolic dysfunction-associated steatohepatitis, chromaturia, flatulence, adverse event, blood glucose increased, and abdominal distension (Supplementary Table S6). These terms should not be interpreted uniformly as new drug-related adverse reactions. Some are likely administrative or non-specific reporting terms, whereas others may represent underlying liver disease, the metabolic background, or clinical manifestations that overlap with labeled hepatobiliary events (Rinella et al., 2023b; U.S. Food and Drug Administration, 2024; Fusaroli et al., 2026).

### 3.4 Reporter-type sensitivity analysis

**FIGURE 4:**
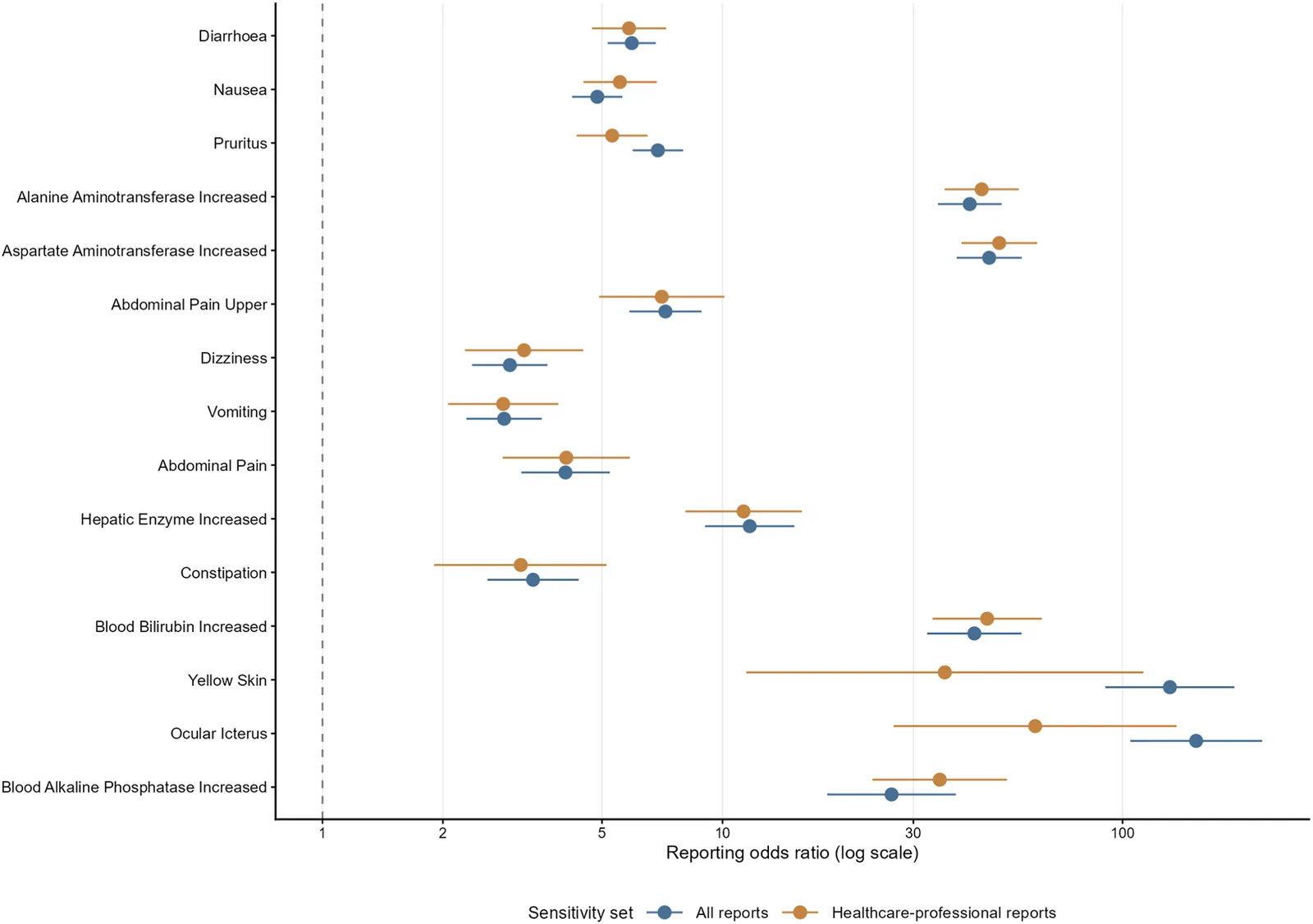
Healthcare professional reporter sensitivity analysis for resmetirom PT signals. ROR estimates for the top resmetirom PT signals are compared between the full reporting set and reports submitted by healthcare professionals. Points indicate RORs, and horizontal lines indicate 95% confidence intervals.

To examine reporter-type effects, we repeated the analysis among healthcare professional reporters only (Figure 4). The HCP-restricted dataset included 1,519,173 FAERS cases, of which 703 were included in the resmetirom single-drug analysis. After restriction to HCP reporters, the leading positive signals changed little from the main analysis. Label-consistent PTs, including pruritus, nausea, diarrhea, alanine aminotransferase increased, aspartate aminotransferase increased, blood bilirubin increased, and hepatic enzyme increased, remained positive. This finding suggests that the principal resmetirom single-drug safety spectrum was not driven solely by non-professional reports, although some unlabeled terms still require cautious clinical interpretation.

### 3.5 Time-to-onset distribution

Among 1,407 reports in the resmetirom single-drug analysis, 313 (22.2%) had valid event-date TTO (Supplementary Figure S1). The median TTO was 31 days (interquartile range, 5–104 days). Events were concentrated early after treatment initiation: 155 reports (49.5% of valid TTO reports) occurred within 0–30 days, followed by 38 (12.1%) within 31–60 days and 36 (11.5%) within 61–90 days. TTO completeness differed by report year, reporter type, and serious outcome status. A valid TTO was available for 74 of 250 reports in 2024 (29.6%), 200 of 846 reports in 2025 (23.6%), and 39 of 311 reports in 2026 (12.5%; *p* < 0.001). It was also more often available in healthcare professional reports than in non-healthcare professional reports (28.0% versus 19.9%; *p* < 0.001) and in serious reports than in non-serious reports (30.5% versus 18.7%; *p* < 0.001) (Supplementary Table S7). TTO availability was not evenly distributed across report year, reporter type, and serious outcome status (Supplementary Table S7). Given the limited availability of TTO data and its uneven distribution across report characteristics, the TTO findings were interpreted descriptively and were not used for causal or interaction inference.

### 3.6 Prespecified resmetirom-statin DDI outcomes

The primary DDI screen did not identify a stable positive interaction pattern for the four prespecified outcomes (Table 3). Focused hepatic injury occurred in 10 of 267 resmetirom–statin co-reports (3.75%) and 24 of 1,162 DDI-resmetirom reports. The odds ratio comparing the co-reporting group with the DDI-resmetirom group was 1.85 (95% CI, 0.87–3.91; *p* = 0.118), and the lower 95% credibility bound of the omega shrinkage statistic was negative (Omega025 = −2.67). Severe hepatic disorders were reported in 23 co-reports (8.61%), with an odds ratio of 1.49 versus DDI-resmetirom reports (95% CI, 0.91–2.44; P = 0.127; Omega025 = −2.49). Gallstone-related disorders were sparse (5 co-reports; 1.87%) and did not show a stable interaction signal (odds ratio, 1.69; 95% CI, 0.60–4.77; *p* = 0.357; Omega025 = −1.23).

**TABLE 3:** Prespecified resmetirom–statin DDI outcomes.

Outcome	A + B events, n/N (%)	DDI-resmetirom cases, N	DDI-statin cases, N	OR (95% CI)	*p*-value	Omega025	IC_025_	RERI	AP	SI	Interpretation
Focused hepatic injury	10/267 (3.75%)	1,162	29,989	1.85 (0.87–3.91)	0.118	−2.67	1.41	−2.76	−0.44	0.66	No stable DDI signal
Severe hepatic disorders	23/267 (8.61%)	1,162	29,989	1.49 (0.91–2.44)	0.127	−2.49	2.06	−2.31	−0.30	0.74	No stable DDI signal
Gallstone-related disorders	5/267 (1.87%)	1,162	29,989	1.69 (0.60–4.77)	0.357	−1.23	0.89	2.19	0.32	1.59	No stable DDI signal
Rhabdomyolysis/myopathy	5/267 (1.87%)	1,162	29,989	11.07 (2.14–57.37)	0.003	−2.98	0.98	−34.13	−4.45	0.16	Sparse and discordant; No stable DDI signal

A + B denotes co-reported resmetirom and statin reports. Prespecified DDI outcomes were evaluated using odds ratios, Omega/IC co-reporting metrics, and additive-interaction indices. Omega025 values below zero indicate no stable evidence of excess co-reporting, not a protective effect.

Rhabdomyolysis/myopathy was also sparse (5 co-reports; 1.87%). Although the odds ratio versus the DDI-resmetirom group was elevated (11.07; 95% CI, 2.14–57.37; *p* = 0.003), the omega statistic did not support a stable co-reporting interaction (Omega025 = −2.98). Omega025 values below zero were interpreted as no stable evidence of excess co-reporting, not as evidence that resmetirom–statin co-reporting protected against the event (Norén et al., 2008). This discordance indicates that the muscle-toxicity finding is unstable and should be considered hypothesis-generating only.

### 3.7 Exploratory shared-PT candidate screen

After the prespecified DDI analysis, a *post hoc* shared-PT screen identified PTs co-reported across the DDI-resmetirom + statin, DDI-resmetirom, and DDI–statin groups (Supplementary Figure S2; Supplementary Table S8). Thirty-six PTs had Omega025 values greater than zero. The highest-ranking candidates included therapy interrupted, death, platelet count decreased, high-density lipoprotein decreased, pollakiuria, cholecystitis, alanine aminotransferase decreased, aspartate aminotransferase decreased, urosepsis, pain, myalgia, and musculoskeletal chest pain. Many terms represented administrative terms, serious outcome terms, background disease manifestations, or sparse event patterns and were interpreted as supplementary candidate signals requiring clinical review.

## 4 Discussion

This FAERS analysis extends early post-marketing evidence for resmetirom through 2026 Q1 and evaluates statin co-reporting in the same cleaned dataset. The resmetirom single-drug signal spectrum was centered on gastrointestinal symptoms, pruritus/skin-related terms, hepatobiliary manifestations, and laboratory abnormalities. This pattern is consistent with the prescribing information and with phase 2 and phase 3 trial findings, in which diarrhea and nausea were the main excess treatment-emergent adverse events and serious adverse events were similar across trial groups (Harrison et al., 2019; Harrison et al., 2023; Harrison et al., 2024; U.S. Food and Drug Administration, 2024). It is also in line with the first published FAERS assessment of resmetirom after approval and with early real-world clinical experience reports, which have begun to describe tolerability and monitoring issues outside trial settings (Kudaravalli et al., 2025; Ravela et al., 2025).

The supplementary SOC-level disproportionality analysis identified Gastrointestinal Disorders, Investigations, Skin And Subcutaneous Tissue Disorders, and Hepatobiliary Disorders, which corresponded to the principal symptom and laboratory patterns observed at the PT level. SOC-level estimates provide a macroscopic summary, but PT-level interpretation remains primary because each SOC combines clinically heterogeneous events and may obscure specific reporting patterns.

The reporter-type sensitivity analysis addressed one practical concern in early FAERS data: whether consumer reports were driving the main signals. The leading gastrointestinal, pruritus, and hepatobiliary/laboratory PTs persisted when both the resmetirom numerator and the FAERS background denominator were restricted to healthcare professional reports. This result supports the main descriptive safety pattern, while background-disease terms such as hepatic cirrhosis and MASH remain less suitable for interpretation as adverse reactions (Bate and Evans, 2009; Rinella et al., 2023b; Fusaroli et al., 2026; U.S. Food and Drug Administration, 2026).

The single-drug PT table separates clinically different types of signals. Label-consistent terms included gastrointestinal symptoms, pruritus, and liver test abnormalities. Some unlabeled PTs were clinically interpretable but still required cautious annotation. Weight decreased, for example, may reflect treatment-related metabolic or weight change, background disease management, reporting behavior, or an adverse event report and should not be interpreted in isolation as either toxicity or treatment benefit. Abdominal discomfort, flatulence, and abdominal distension may represent more granular gastrointestinal reporting as post-marketing use expands. Chromaturia appeared in the complete positive PT table rather than in the top 20 PT table (Supplementary Table S6; 22 reports), but it has limited mechanistic support from the current label or clinical trial safety profile. We, therefore, treated chromaturia as a lower-priority surveillance term unless supported by accumulating cases or more detailed clinical narratives. Other PTs, including therapy interrupted, unevaluable event, disease progression, hepatic cirrhosis, and metabolic dysfunction-associated steatohepatitis, are better read as treatment-course, administrative, or background-disease reports than as candidate adverse reactions (Rinella et al., 2023b; Fusaroli et al., 2026).

The resmetirom–statin analysis is clinically relevant because lipid-lowering treatment is common in the cardiometabolic population for whom resmetirom is prescribed. Statin safety in chronic liver disease has been addressed in clinical guidance and observational evidence, and the resmetirom label provides dose recommendations for several statins because resmetirom can increase statin exposure (Targher et al., 2016; Newman et al., 2019; Pastori et al., 2022; Rinella et al., 2023b). In our suspect-anchored FAERS analysis, hepatic and gallbladder-related outcomes did not show stable interaction-specific disproportionality. Rhabdomyolysis/myopathy was based on five co-reports and showed a discordant pattern: an elevated odds ratio but a negative omega lower bound. This is a typical sparse-data scenario in which shrinkage-based interaction metrics are more informative than crude odds ratios alone (Norén et al., 2008; Jiao et al., 2024).

The shared-PT screen was useful as a secondary check but did not alter the DDI interpretation. Candidate PTs appeared across the DDI-resmetirom, DDI–statin, and DDI-resmetirom + statin groups, including treatment-modification terms, serious outcome terms, hepatobiliary/metabolic terms, and sparse muscle-related reports. Most candidates had small co-report counts or non-specific PT semantics. The screen, therefore, served mainly to verify that no obvious shared PT cluster was being missed by the four prespecified DDI outcomes; it did not identify a new outcome category that would change the primary DDI interpretation (Zhang et al., 2024).

The drug-role definitions were chosen to match the purpose of each analysis. The single-drug analysis required resmetirom to be recorded as a suspect drug, which limits dilution from incidental concomitant drugs. The co-reporting analysis allowed resmetirom and statins to appear in any drug role within the same case, provided that at least one of the two drugs was recorded as PS or SS. This suspect-anchored definition kept potential interaction reports in which the second drug was coded as concomitant, while avoiding a fully unrestricted co-medication screen. Similar trade-offs are common in spontaneous-reporting interaction studies, where stricter role-code rules reduce noise but may miss plausible interaction reports (Norén et al., 2008; Fusaroli et al., 2024; Jiao et al., 2024).

Several limitations define the boundary of these results. The resmetirom-containing groups were entirely U.S.-based, reflecting early U.S. access, prescribing, and spontaneous-reporting practices after approval. The signal profile may, therefore, not generalize directly to healthcare systems with different reimbursement, prescribing, or pharmacovigilance patterns. Demographic data were incomplete, with age available for only 10.5% of reports in the resmetirom single-drug analysis. This limited completeness reduces the generalizability of the descriptive patient profile and precludes reliable age-stratified interpretation. If completeness differs by reporter type, seriousness, or report quality, descriptive patient summaries and signal ordering may be affected. FAERS cannot estimate incidence, absolute risk, or causality, and reporting can be affected by under-reporting, stimulated reporting, missing information, and duplicate or incomplete submissions despite deduplication (Bate and Evans, 2009; Harpaz et al., 2012; Sakaeda et al., 2013; U.S. Food and Drug Administration, 2026). Sex, age, dosing, treatment duration, liver fibrosis stage, baseline liver tests, statin dose, and laboratory trajectories were unavailable or incomplete. TTO was available for only 22.2% of reports and was more frequently documented in healthcare professional and serious reports. The subset with calculable TTO may, therefore, not represent the full reporting population, and chronic or delayed-onset events may be underrepresented. TTO findings were consequently interpreted as descriptive temporal patterns rather than evidence of temporal causality (Van Holle et al., 2012; Sauzet et al., 2013). Confounding by indication remains a central limitation of the resmetirom–statin analysis. MASH severity, dyslipidemia, baseline liver abnormalities, healthcare-contact intensity, and co-medication burden may influence both statin exposure and adverse event reporting. The A + B, A-only, B-only, and neither groups should, therefore, be interpreted as co-reporting strata rather than comparable treatment cohorts (Targher et al., 2016; Rinella et al., 2023b; Fusaroli et al., 2026). FAERS does not provide treatment denominators, lipid profiles, baseline liver tests, fibrosis stage, drug doses, longitudinal laboratory trajectories, or untreated comparator risk sets. These data cannot adequately separate a pharmacological interaction from differences in indication, disease severity, or reporting structure. The DDI results, therefore, indicate that no stable spontaneous-reporting interaction signal was detected for the prespecified outcomes; they do not establish the absence of clinical risk during resmetirom–statin co-use. The small number of co-reported target events further limits the inference of drug interactions.

These issues are not unique to resmetirom; they reflect the causal structure of spontaneous reporting data. Directed acyclic graph approaches to pharmacovigilance emphasize that disease severity, treatment selection, healthcare contact, and reporting behavior can influence both exposure and outcome recording (Fusaroli et al., 2026). For this reason, the value of the present analysis lies in signal ordering, clinical annotation, and prioritization for follow-up, not in estimating the causal effect of resmetirom or resmetirom–statin co-exposure.

Taken together, these findings support three surveillance priorities: continued monitoring of labeled gastrointestinal, pruritus, and hepatobiliary/laboratory events; clinical review of unlabeled but interpretable PTs as report numbers increase; and continued pharmacovigilance for resmetirom–statin co-reporting, with emphasis on hepatic, gallbladder-related, and muscle-related outcomes in larger real-world datasets.

## 5 Conclusion

In FAERS reports from 2024 Q1 to 2026 Q1, the resmetirom single-drug safety spectrum was mainly composed of gastrointestinal events, pruritus/skin-related terms, and hepatobiliary laboratory or clinical terms, consistent with the FDA label and clinical trial evidence. Several unlabeled PTs appeared among the top signals, including weight decreased and abdominal discomfort, whereas administrative and background-disease terms require clinical review before being treated as adverse reactions. Exploratory resmetirom–statin co-reporting analyses did not demonstrate stable interaction-specific hepatic, gallbladder-related, or muscle-related toxicity signals. These results provide an early post-marketing reference for resmetirom surveillance as its use expands beyond trial populations.

## Data Availability

The original contributions presented in the study are included in the article/Supplementary Material; further inquiries can be directed to the corresponding author.

## References

[B1] Barrett T. Dowle M. Srinivasan A. Gorecki J. Chirico M. Hocking T. (2026). data.table: Extension of ‘data.frame’. Vienna, Austria: The R Foundation. 10.32614/CRAN.package.data.table

[B2] Bate A. Evans S. J. W. (2009). Quantitative signal detection using spontaneous ADR reporting. Pharmacoepidemiol. Drug Saf. 18, 427–436. 10.1002/pds.1742 19358225

[B3] Bate A. Lindquist M. Edwards I. R. Olsson S. Orre R. Lansner A. (1998). A Bayesian neural network method for adverse drug reaction signal generation. Eur. J. Clin. Pharmacol. 54, 315–321. 10.1007/s002280050466 9696956

[B4] Brown E. G. Wood L. Wood S. (1999). The medical dictionary for regulatory activities (MedDRA). Drug Saf. 20, 109–117. 10.2165/00002018-199920020-00002 10082069

[B5] Chalasani N. P. Maddur H. Russo M. W. Wong R. J. Reddy K. R. On Behalf Of The Practice Parameters Committee Of The American College Of Gastroenterology (2021). ACG clinical guideline: diagnosis and management of idiosyncratic drug-induced liver injury. Am. J. Gastroenterol. 116, 878–898. 10.14309/ajg.0000000000001259 33929376

[B6] Evans S. J. W. Waller P. C. Davis S. (2001). Use of proportional reporting ratios (PRRs) for signal generation from spontaneous adverse drug reaction reports. Pharmacoepidemiol. Drug Saf. 10, 483–486. 10.1002/pds.677 11828828

[B7] Fusaroli M. Salvo F. Begaud B. AlShammari T. M. Bate A. Battini V. (2024). The REporting of a disproportionality analysis for DrUg safety signal detection using individual case safety reports in PharmacoVigilance (READUS-PV): explanation and elaboration. Drug Saf. 47, 585–599. 10.1007/s40264-024-01423-7 38713347 PMC11116264

[B8] Fusaroli M. Mitchell J. Rudolph A. Rocca E. Fusaroli R. (2026). Causal inference tools for pharmacovigilance: using causal graphs to identify and address biases in disproportionality analysis. Drug Saf. 49, 449–469. 10.1007/s40264-025-01628-4 41381798 PMC13002730

[B9] Gohel D. Skintzos P. (2026). Flextable: Functions for Tabular Reporting. 10.32614/CRAN.package.flextable

[B10] Gohel D. Moog S. Heckmann M. (2026). Officer: Manipulation of Microsoft Word and Powerpoint Documents. 10.32614/CRAN.package.officer

[B11] Harpaz R. DuMouchel W. Shah N. H. Madigan D. Ryan P. Friedman C. (2012). Novel data-mining methodologies for adverse drug event discovery and analysis. Clin. Pharmacol. Ther. 91, 1010–1021. 10.1038/clpt.2012.50 22549283 PMC3675775

[B12] Harrison S. A. Bashir M. R. Guy C. D. Zhou R. Moylan C. A. Frias J. P. (2019). Resmetirom (MGL-3196) for the treatment of non-alcoholic steatohepatitis: a multicentre, randomised, double-blind, placebo-controlled, phase 2 trial. Lancet 394, 2012–2024. 10.1016/S0140-6736(19)32517-6 31727409

[B13] Harrison S. A. Taub R. Neff G. W. Lucas K. J. Labriola D. Moussa S. E. (2023). Resmetirom for nonalcoholic fatty liver disease: a randomized, double-blind, placebo-controlled phase 3 trial. Nat. Med. 29, 2919–2928. 10.1038/s41591-023-02603-1 37845512 PMC10667098

[B14] Harrison S. A. Bedossa P. Guy C. D. Schattenberg J. M. Loomba R. Taub R. (2024). A phase 3, randomized, controlled trial of resmetirom in NASH with liver fibrosis. N. Engl. J. Med. 390, 497–509. 10.1056/NEJMoa2309000 38324483

[B15] Jiao X. Pu L. Lan S. Li H. Zeng L. Wang H. (2024). Adverse drug reaction signal detection methods in spontaneous reporting system: a systematic review. Pharmacoepidemiol. Drug Saf. 33, e5768. 10.1002/pds.5768 38419132

[B16] Kudaravalli P. Andrews M. B. Adler D. G. (2025). An analysis of safety data from the first year of resmetirom. Gastroenterol. Rep. 13, goaf097. 10.1093/gastro/goaf097 41230405 PMC12603349

[B17] MedDRA Maintenance And Support Services Organization (2025). Introductory guide for standardised MedDRA queries (SMQs). Available online at: https://www.meddra.org/how-to-use/support-documentation (Accessed August 8, 2026).

[B18] Newman C. B. Preiss D. Tobert J. A. Jacobson T. A. Page R. L. Goldstein L. B. (2019). Statin safety and associated adverse events: a scientific statement from the American heart association. Arterioscler. Thromb. Vasc. Biol. 39, 1–44. 10.1161/ATV.0000000000000073 30580575

[B19] Norén G. N. Sundberg R. Bate A. Edwards I. R. (2008). A statistical methodology for drug–drug interaction surveillance. Statistics Med. 27, 3057–3070. 10.1002/sim.3247 18344185

[B20] Pastori D. Pani A. Di Rocco A. Menichelli D. Gazzaniga G. Farcomeni A. (2022). Statin liver safety in non‐alcoholic fatty liver disease: a systematic review and metanalysis. Br. J. Clin. Pharmacol. 88, 441–451. 10.1111/bcp.14943 34133035 PMC9290532

[B21] R Core Team (2026). R: A Language and Environment for Statistical Computing. Vienna, Austria: R Foundation for Statistical Computing. Available online at: https://www.R-project.org/ (Accessed August 8, 2026).

[B22] Ravela N. Shackelford P. Blessing N. Yoder L. Chalasani N. Samala N. (2025). Early experience with resmetirom to treat metabolic dysfunction–associated steatohepatitis with fibrosis in a real-world setting. Hepatol. Commun. 9, e0670. 10.1097/HC9.0000000000000670 40178494 PMC11970881

[B23] Rinella M. E. Lazarus J. V. Ratziu V. Francque S. M. Sanyal A. J. Kanwal F. (2023a). A multisociety Delphi consensus statement on new fatty liver disease nomenclature. Hepatology 78, 1966–1986. 10.1097/HEP.0000000000000520 37363821 PMC10653297

[B24] Rinella M. E. Neuschwander-Tetri B. A. Siddiqui M. S. Abdelmalek M. F. Caldwell S. Barb D. (2023b). AASLD practice guidance on the clinical assessment and management of nonalcoholic fatty liver disease. Hepatology 77, 1797–1835. 10.1097/HEP.0000000000000323 36727674 PMC10735173

[B25] Sakaeda T. Tamon A. Kadoyama K. Okuno Y. (2013). Data mining of the public version of the FDA adverse event reporting System. Int. J. Med. Sci. 10, 796–803. 10.7150/ijms.6048 23794943 PMC3689877

[B26] Sauzet O. Carvajal A. Escudero A. Molokhia M. Cornelius V. R. (2013). Illustration of the weibull shape parameter signal detection tool using electronic healthcare record data. Drug Saf. 36, 995–1006. 10.1007/s40264-013-0061-7 23673816

[B27] Schauberger P. Walker A. (2025). Openxlsx: Read, Write and Edit Xlsx Files. 10.32614/CRAN.package.openxlsx

[B28] Targher G. Byrne C. D. Lonardo A. Zoppini G. Barbui C. (2016). Non-alcoholic fatty liver disease and risk of incident cardiovascular disease: a meta-analysis. J. Hepatol. 65, 589–600. 10.1016/j.jhep.2016.05.013 27212244

[B29] U.S. Food And Drug Administration (2024). Rezdiffra (Resmetirom) Prescribing Information. Silver Spring, MD: U.S. Food and Drug Administration. Available online at: https://www.accessdata.fda.gov/drugsatfda_docs/label/2024/217785s000lbl.pdf (Accessed August 8, 2026).

[B30] U.S. Food And Drug Administration (2026). FAERS quarterly data extract files. Available online at: https://fis.fda.gov/extensions/FPD-QDE-FAERS/FPD-QDE-FAERS.html (Accessed August 8, 2026).

[B31] Van Holle L. Zeinoun Z. Bauchau V. Verstraeten T. (2012). Using time to onset for detecting safety signals in spontaneous reports of adverse events following immunization: a proof of concept study. Pharmacoepidemiol. Drug Saf. 21, 603–610. 10.1002/pds.3226 22383219

[B32] Van Puijenbroek E. P. Bate A. Leufkens H. G. M. Lindquist M. Orre R. Egberts A. C. G. (2002). A comparison of measures of disproportionality for signal detection in spontaneous reporting systems for adverse drug reactions. Pharmacoepidemiol. Drug Saf. 11, 3–10. 10.1002/pds.668 11998548

[B33] Wickham H. (2016). ggplot2: Elegant Graphics for Data Analysis. New York: Springer-Verlag. Available online at: https://ggplot2.tidyverse.org (Accessed August 8, 2026).

[B34] Younossi Z. M. Koenig A. B. Abdelatif D. Fazel Y. Henry L. Wymer M. (2016). Global epidemiology of nonalcoholic fatty liver disease—Meta-analytic assessment of prevalence, incidence, and outcomes. Hepatology 64, 73–84. 10.1002/hep.28431 26707365

[B35] Zhang S. Yan M.-M. Zhao H. Qiu X.-Y. Zhu D. (2024). Rhabdomyolysis associated with concomitant use of colchicine and statins in the real world: identifying the likelihood of drug–drug interactions through the FDA adverse event reporting system. Front. Pharmacol. 15, 1445324. 10.3389/fphar.2024.1445324 39351090 PMC11439674

