# Supplementary material for: Early post-marketing safety profile of resmetirom in FAERS: an exploratory assessment of statin co-reporting
Source: Front Pharmacol. 2026 Aug 20;17:1895524. doi: 10.3389/fphar.2026.1895524 (PMC13538014; doi:10.3389/fphar.2026.1895524)
Supplement: Supplementary file 1 [file Supplementaryfile1.docx]

**Supplemental Figures**

**Supplementary Figure S1.** Time-to-onset distribution in the resmetirom single-drug analysis. Horizontal bars show event-date-based time-to-onset (TTO) among reports in the resmetirom single-drug analysis with valid drug start and event onset dates. Labels show report counts and percentages among valid TTO reports. TTO was available for 313 of 1,407 reports (22.2%); median TTO was 31 days (interquartile range, 5-104 days).

**Supplementary Figure S2.** Post hoc shared-PT screen across the DDI-resmetirom, DDI-statin and DDI-resmetirom+statin groups. Points represent candidate PTs and are sized by the number of A+B reports. This analysis was exploratory and hypothesis-generating.

**Supplemental Table**

**Supplementary Table S1.** Drug-name search terms used in this study.

| **Drug class** | **Search term** | **Role in analysis** |
| --- | --- | --- |
| Resmetirom | RESMETIROM | A drug |
| Resmetirom | REZDIFFRA | A drug |
| Resmetirom | MGL-3196 | A drug |
| Resmetirom | MGL3196 | A drug |
| Statin generic | ATORVASTATIN | B drug |
| Statin generic | ROSUVASTATIN | B drug |
| Statin generic | SIMVASTATIN | B drug |
| Statin generic | PRAVASTATIN | B drug |
| Statin generic | LOVASTATIN | B drug |
| Statin generic | FLUVASTATIN | B drug |
| Statin generic | PITAVASTATIN | B drug |
| Statin generic | CERIVASTATIN | B drug |
| Statin brand/combo brand | LIPITOR | B drug |
| Statin brand/combo brand | ATORVALIQ | B drug |
| Statin brand/combo brand | CADUET | B drug |
| Statin brand/combo brand | CRESTOR | B drug |
| Statin brand/combo brand | EZALLOR | B drug |
| Statin brand/combo brand | ROSZET | B drug |
| Statin brand/combo brand | ZOCOR | B drug |
| Statin brand/combo brand | FLOLIPID | B drug |
| Statin brand/combo brand | VYTORIN | B drug |
| Statin brand/combo brand | SIMCOR | B drug |
| Statin brand/combo brand | JUVISYNC | B drug |
| Statin brand/combo brand | PRAVACHOL | B drug |
| Statin brand/combo brand | MEVACOR | B drug |
| Statin brand/combo brand | ALTOPREV | B drug |
| Statin brand/combo brand | ADVICOR | B drug |
| Statin brand/combo brand | LESCOL | B drug |
| Statin brand/combo brand | LIVALO | B drug |
| Statin brand/combo brand | ZYPITAMAG | B drug |
| Statin brand/combo brand | LIVAZO | B drug |
| Statin brand/combo brand | BAYCOL | B drug |
| Statin brand/combo brand | LIPOBAY | B drug |

**Supplementary Table S2.** Two-by-two contingency table for disproportionality analysis.

| **Item** | **Target adverse events reported** | **Other adverse events reported** | **Total** |
| --- | --- | --- | --- |
| Target drugs | a | b | a + b |
| Other drugs | c | d | c + d |
| Total | a + c | b + d | a + b + c + d |

**Supplementary Table S3.** Formulae and interpretation thresholds for signal-detection and DDI metrics.

| **Method** | **Calculation formula** | **Criteria** |
| --- | --- | --- |
| ROR | $\text{ROR=}\frac{\text{a / c}}{\text{b}\text{ / }\text{d}}$ | a ≥ 3  95%CI (lower limit) > 1 |
|  | $\text{SE}\text{(}\text{lnROR}\text{)=}\sqrt{\frac{\text{1}}{\text{a}}\text{+}\frac{\text{1}}{\text{b}}\text{+}\frac{\text{1}}{\text{c}}\text{+}\frac{\text{1}}{\text{d}}}$ |  |
|  | $\text{95\%}\text{CI}\text{= }\text{e}^{\ln\left( \text{ROR} \right)\text{±1.96}\text{se}}$ |  |
| PRR | $\text{PRR=}\frac{\text{a / (a+b)}}{\text{c}\text{ / (}\text{c}\text{+}\text{d}\text{)}}$ | a ≥ 3  PRR ≥ 2  $\text{χ}$^2^ ≥ 4 |
|  | $\text{χ2 =}\frac{\text{ }\text{(ad−bc)}^{\text{2}}\text{(a+b+c+d)}}{\text{( a+b)(a+c)(c+d)(b+d)}}$ |  |
| IC/BCPNN | IC=$\text{log}_{\text{2}}\frac{\text{p}\text{(}\text{x}\text{,}\text{y}\text{)}}{\text{p}\text{(}\text{x}\text{)}\text{p}\text{(}\text{y}\text{)}}\text{=}\text{log}_{\text{2}}\frac{\text{a}\text{(}\text{a}\text{+}\text{b}\text{+}\text{c}\text{+}\text{d}\text{)}}{\text{(}\text{a}\text{+}\text{b}\text{)(}\text{a}\text{+}\text{c}\text{)}}$ | IC025>0 |
|  | E(IC)=$\text{log}_{\text{2}}\frac{\text{(}\text{a}\text{+}\text{γ}\text{11)(}\text{a}\text{+}\text{b}\text{+}\text{c}\text{+}\text{d}\text{+}\text{α}\text{)(}\text{a}\text{+}\text{b}\text{+}\text{c}\text{+}\text{d}\text{+}\text{β}\text{)}}{\text{（}\text{a}\text{+}\text{b}\text{+}\text{c}\text{+}\text{d}\text{+}\text{γ}\text{）(}\text{a}\text{+}\text{b}\text{+}\text{α}\text{1)(}\text{a}\text{+}\text{c}\text{+}\text{β}\text{1)}}$ |  |
|  | $\text{V}\left( \text{IC} \right)\text{=}\frac{\text{1}}{{\text{(}\text{ln}\text{2)}}^{\text{2}}}\text{\{}\left[ \frac{\left( \text{a}\text{+}\text{b}\text{+}\text{c}\text{+}\text{d} \right)\text{−}\text{a}\text{+}\text{γ}\text{−}\text{γ}\text{11}}{\left( \text{a}\text{+}\text{γ}\text{11} \right)\left( \text{1+}\text{a}\text{+}\text{b}\text{+}\text{c}\text{+}\text{d}\text{+}\text{γ} \right)} \right]\text{+}$  $\left[ \frac{\left( \text{a}\text{+}\text{b}\text{+}\text{c}\text{+}\text{d} \right)\text{−}\left( \text{a}\text{+}\text{b} \right)\text{+}\text{α}\text{−}\text{α}\text{1}}{\left( \text{a}\text{+}\text{b}\text{+}\text{α}\text{1} \right)\left( \text{1+}\text{a}\text{+}\text{b}\text{+}\text{c}\text{+}\text{d}\text{+}\text{α} \right)} \right]\text{+}\left[ \frac{\left( \text{a}\text{+}\text{b}\text{+}\text{c}\text{+}\text{d} \right)\text{−}\left( \text{a}\text{+}\text{c} \right)\text{+}\text{β}\text{−}\text{β}\text{1}}{\left( \text{a}\text{+}\text{c}\text{+}\text{β}\text{1} \right)\left( \text{1+}\text{a}\text{+}\text{b}\text{+}\text{c}\text{+}\text{d}\text{+}\text{β} \right)} \right]\text{\}}$ |  |
|  | $\text{γ}\text{=}\text{γ}\text{11}\frac{\text{(}\text{a}\text{+}\text{b}\text{+}\text{c}\text{+}\text{d}\text{+}\text{α}\text{)(}\text{a}\text{+}\text{b}\text{+}\text{c}\text{+}\text{d}\text{+}\text{β}\text{)}}{\text{(}\text{a}\text{+}\text{b}\text{+}\text{α}\text{1)(}\text{a}\text{+}\text{c}\text{+}\text{β}\text{1)}}$ |  |
|  | *IC-2SD=E(IC)-2*$\sqrt{\text{V}\text{(}\text{IC}\text{)}}$  $\text{α}\text{1=}\text{β}\text{1=1；}\text{α}\text{=}\text{β}\text{=2；}\text{γ}\text{11=1}$ |  |
|  | $\text{SE}\text{(}\text{lnEBGM}\text{)=}\sqrt{\frac{\text{1}}{\text{a}}\text{+}\frac{\text{1}}{\text{b}}\text{+}\frac{\text{1}}{\text{c}}\text{+}\frac{\text{1}}{\text{d}}}$ |  |
|  | $\text{95\%}\text{CI}\text{= }\text{e}^{\ln\left( \text{EBGM} \right)\text{±1.96}\text{se}}$ |  |
| Omega | Omega = log2[(n111 + 0.5)/(E111 + 0.5)], where E111 is the expected number of A+B outcome reports under multiplicative independence of A and B | Omega025 below zero indicates no stable evidence of excess co-reporting |
| Pairwise IC | Pairwise IC = log2[(observed + 0.5)/(expected + 0.5)] for the drug-pair/outcome co-reporting table | Supportive co-reporting metric for the drug pair and outcome |
| RERI | RERI = OR11 - OR10 - OR01 + 1 | Additive interaction metric; values above zero suggest excess risk on the additive scale |
| AP | AP = RERI/OR11 | Proportion of OR11 attributable to additive interaction |
| SI | SI = (OR11 - 1)/[(OR10 - 1) + (OR01 - 1)] | Additive interaction index; values above one suggest positive interaction on the additive scale |

For DDI metrics, A denotes resmetirom and B denotes a statin.

**Supplementary Table S4.** SOC-level disproportionality results for resmetirom single-drug reports.

| **SOC** | **Resmetirom reports, n** | **All other FAERS reports, n** | **ROR**  **(95% CI)** | **PRR** | **Chi-square** | **IC** | **IC025** | **ROR positive** | **PRR positive** | **IC positive** | **Positive algorithms** |
| --- | --- | --- | --- | --- | --- | --- | --- | --- | --- | --- | --- |
| Gastrointestinal Disorders | 530 | 479,573 | 3.37 (3.03-3.75) | 2.48 | 549.99 | 1.31 | 1.18 | Yes | Yes | Yes | 3/3 |
| Investigations | 430 | 353,037 | 3.49 (3.12-3.91) | 2.73 | 530.25 | 1.44 | 1.31 | Yes | Yes | Yes | 3/3 |
| Skin And Subcutaneous Tissue Disorders | 333 | 370,831 | 2.33 (2.06-2.63) | 2.01 | 192.14 | 1.01 | 0.85 | Yes | Yes | Yes | 3/3 |
| Hepatobiliary Disorders | 255 | 77,921 | 8.74 (7.63-10.01) | 7.34 | 1425.99 | 2.85 | 2.68 | Yes | Yes | Yes | 3/3 |
| General Disorders And Administration Site Conditions | 444 | 1,171,152 | 0.78 (0.70-0.87) | 0.85 | 18.76 | -0.23 | -0.37 | No | No | No | 0/3 |
| Nervous System Disorders | 209 | 479,934 | 0.97 (0.84-1.13) | 0.98 | 0.14 | -0.03 | -0.23 | No | No | No | 0/3 |
| Injury, Poisoning And Procedural Complications | 128 | 997,586 | 0.22 (0.18-0.26) | 0.29 | 330.31 | -1.79 | -2.04 | No | No | No | 0/3 |
| Musculoskeletal And Connective Tissue Disorders | 80 | 289,694 | 0.60 (0.48-0.75) | 0.62 | 20.66 | -0.69 | -1.00 | No | No | No | 0/3 |
| Surgical And Medical Procedures | 76 | 155,134 | 1.10 (0.88-1.39) | 1.10 | 0.70 | 0.13 | -0.19 | No | No | No | 0/3 |
| Psychiatric Disorders | 74 | 270,669 | 0.59 (0.47-0.75) | 0.61 | 19.80 | -0.70 | -1.03 | No | No | No | 0/3 |
| Infections And Infestations | 65 | 390,711 | 0.34 (0.27-0.44) | 0.37 | 78.23 | -1.42 | -1.77 | No | No | No | 0/3 |
| Metabolism And Nutrition Disorders | 62 | 160,628 | 0.86 (0.67-1.11) | 0.87 | 1.37 | -0.21 | -0.57 | No | No | No | 0/3 |
| Renal And Urinary Disorders | 50 | 121,197 | 0.92 (0.70-1.22) | 0.92 | 0.32 | -0.11 | -0.51 | No | No | No | 0/3 |
| Respiratory, Thoracic And Mediastinal Disorders | 40 | 299,287 | 0.28 (0.20-0.38) | 0.30 | 72.37 | -1.73 | -2.17 | No | No | No | 0/3 |
| Immune System Disorders | 30 | 107,496 | 0.62 (0.43-0.89) | 0.63 | 6.96 | -0.67 | -1.18 | No | No | No | 0/3 |
| Eye Disorders | 30 | 147,972 | 0.44 (0.31-0.64) | 0.45 | 20.61 | -1.12 | -1.64 | No | No | No | 0/3 |
| Neoplasms Benign, Malignant And Unspecified (Incl Cysts And Polyps) | 19 | 144,185 | 0.29 (0.18-0.45) | 0.30 | 33.47 | -1.73 | -2.38 | No | No | No | 0/3 |
| Cardiac Disorders | 19 | 144,405 | 0.29 (0.18-0.45) | 0.29 | 33.56 | -1.73 | -2.38 | No | No | No | 0/3 |
| Blood And Lymphatic System Disorders | 15 | 138,477 | 0.23 (0.14-0.39) | 0.24 | 37.05 | -2.01 | -2.74 | No | No | No | 0/3 |
| Vascular Disorders | 15 | 159,918 | 0.20 (0.12-0.34) | 0.21 | 46.87 | -2.21 | -2.94 | No | No | No | 0/3 |
| Social Circumstances | 10 | 46,551 | 0.48 (0.26-0.89) | 0.48 | 5.67 | -1.02 | -1.91 | No | No | No | 0/3 |
| Reproductive System And Breast Disorders | 8 | 47,426 | 0.37 (0.19-0.75) | 0.38 | 8.31 | -1.35 | -2.35 | No | No | No | 0/3 |
| Ear And Labyrinth Disorders | 6 | 34,763 | 0.38 (0.17-0.86) | 0.39 | 5.89 | -1.30 | -2.45 | No | No | No | 0/3 |
| Endocrine Disorders | 5 | 26,342 | 0.42 (0.18-1.02) | 0.43 | 3.91 | -1.16 | -2.42 | No | No | No | 0/3 |
| Product Issues | 4 | 184,347 | 0.05 (0.02-0.12) | 0.05 | 79.06 | -4.20 | -5.61 | No | No | No | 0/3 |
| Unmapped | 0 | 3,536 | 0.32 (0.02-5.06) | 0.32 | 0.74 | -1.06 | -5.05 | No | No | No | 0/3 |
| Congenital, Familial And Genetic Disorders | 0 | 18,458 | 0.06 (0.00-0.97) | 0.06 | 7.31 | -3.13 | -7.13 | No | No | No | 0/3 |
| Pregnancy, Puerperium And Perinatal Conditions | 0 | 22,477 | 0.05 (0.00-0.79) | 0.05 | 9.13 | -3.40 | -7.40 | No | No | No | 0/3 |

SOC-level disproportionality was calculated using the same case-level background and positivity thresholds as the PT-level analysis. A positive SOC met all three criteria: ROR lower 95% confidence limit >1; PRR ≥2 with chi-square ≥4; and IC025 >0. IC, information component; PRR, proportional reporting ratio; ROR, reporting odds ratio; SOC, system organ class.

**Supplementary Table S5.**  PT definitions for prespecified DDI outcomes.

The DDI outcomes were prespecified from the FDA label, known statin safety concerns, mechanistic plausibility and MedDRA/SMQ-based groupings.

In Data Sheet-Table S5 sheet.

**Supplementary Table S6.**  All positive PT signals in the resmetirom single-drug analysis.

In Data Sheet-Table S6 sheet.

**Supplementary Table S7.** Completeness of event-date-based time-to-onset data in the resmetirom single-drug analysis.

| **Domain** | **Category** | **Total reports, n** | **TTO available, n (%)** | **TTO unavailable, n (%)** | **Median TTO among available reports, days [IQR]** | **P value for TTO availability** |
| --- | --- | --- | --- | --- | --- | --- |
| **Overall** | Overall | 1,407 | 313 (22.2%) | 1,094 (77.8%) | 31 [5, 104] |  |
| Report year | 2024 | 250 | 74 (29.6%) | 176 (70.4%) | 13 [2, 48] | <0.001 |
| Report year | 2025 | 846 | 200 (23.6%) | 646 (76.4%) | 36 [5, 108] |  |
| Report year | 2026 | 311 | 39 (12.5%) | 272 (87.5%) | 108 [23, 179] |  |
| Report quarter | 2024Q2 | 2 | 1 (50.0%) | 1 (50.0%) | 17 [17, 17] | <0.001 |
| Report quarter | 2024Q3 | 109 | 28 (25.7%) | 81 (74.3%) | 10 [3, 17] |  |
| Report quarter | 2024Q4 | 139 | 45 (32.4%) | 94 (67.6%) | 17 [1, 61] |  |
| Report quarter | 2025Q1 | 118 | 34 (28.8%) | 84 (71.2%) | 62 [20, 92] |  |
| Report quarter | 2025Q2 | 148 | 39 (26.4%) | 109 (73.6%) | 24 [4, 116] |  |
| Report quarter | 2025Q3 | 324 | 74 (22.8%) | 250 (77.2%) | 32 [4, 106] |  |
| Report quarter | 2025Q4 | 256 | 53 (20.7%) | 203 (79.3%) | 38 [15, 108] |  |
| Report quarter | 2026Q1 | 311 | 39 (12.5%) | 272 (87.5%) | 108 [23, 179] |  |
| Reporter type | Healthcare professional | 703 | 197 (28.0%) | 506 (72.0%) | 43 [11, 108] | <0.001 |
| Reporter type | Non-healthcare professional | 553 | 110 (19.9%) | 443 (80.1%) | 18 [1, 72] |  |
| Reporter type | Missing/other | 151 | 6 (4.0%) | 145 (96.0%) | 61 [18, 68] |  |
| Serious outcome status | Non-serious outcome | 988 | 185 (18.7%) | 803 (81.3%) | 17 [2, 60] | <0.001 |
| Serious outcome status | Serious outcome | 419 | 128 (30.5%) | 291 (69.5%) | 74 [22, 149] |  |

Values are n (%) unless otherwise indicated. TTO was considered available when both the resmetirom start date and event onset date were available and yielded a valid non-negative interval. Healthcare-professional reporters included MD, PH and HP occupation codes; non-healthcare-professional reporters included CN and LW. Serious outcome was defined by at least one FAERS OUTC record coded as DE, HO, LT, DS, RI, OT or CA. P values were calculated using Pearson's chi-square test when all expected cell counts were at least 5; Fisher's exact test was used for sparse 2 × 2 tables, and a chi-square test with 20,000 Monte Carlo replicates was used for larger sparse tables.

**Supplementary Table S8**. Complete exploratory shared-PT candidate screen.

Candidate score was an exploratory prioritisation score ranging from 0 to 5. One point was assigned for each of the following: A+B reports of five or more; OR for A+B versus DDI-resmetirom greater than 1.5 with lower 95% CI greater than 1; OR for A+B versus DDI-statin greater than 1.5 with lower 95% CI greater than 1; Omega025 greater than 0; and pair IC025 greater than 0. The score was used only to rank shared PT candidates and was not a prespecified signal-detection criterion.

In Data Sheet-Table S8 sheet.
